# Supplementary material for: Optimization of Aedes albopictus (Diptera: Culicidae) Mass Rearing through Cost-Effective Larval Feeding
Source: Insects. 2022 May 26;13(6):504. doi: 10.3390/insects13060504 (PMC9224466; doi:10.3390/insects13060504)
Supplement: Supplementary file 1 [file insects-13-00504-s001.zip › insects-1707344-supplementary.pdf]

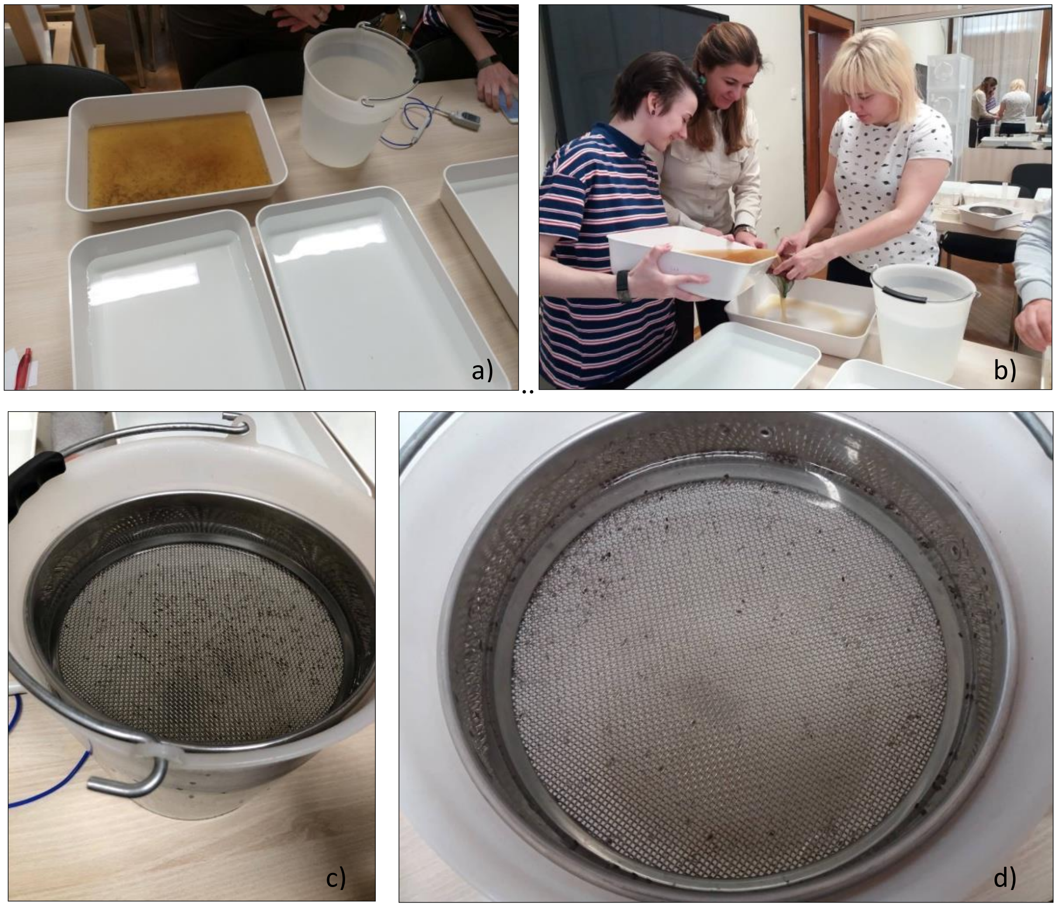

Supplemental Figure S1: Equipment and application of the sieving method for extraction of *Aedes albopictus* male pupae.

a) Equipment prepared before the start of sieving : Rearing tray with juveniles, bucket with tepid water before the placement of the sieve, thermometer to measure the water temperature in the bucket and two trays with water for the forthcoming separation of pupae (one tray is intended for pupae which will pass through the sieve and one for juveniles that will not pass through the sieve);
b) Separation of all juveniles from the rearing water by a dense net (afterward juveniles are transferred from the net into the bucket for sieving);
c) Position of the sieve in the bucket: after the transfer of juveniles into the bucket with water, the sieve is placed in a way that the mesh is submerged 2 cm below the water surface, allowing small size pupae (assumed to be males) to pass through the to the mesh while moving upwards;
d) Sieve with small size pupae which successfully passed through the mesh. The rest of the juveniles (i.e. larger size pupae and larvae) remained in water beneath the sieve. (After the end of sieving (3 min exposure), the sieve with pupae which passed through the

mesh is removed from the bucket and pupae are transferred in a tray with clean water, while juveniles which did not pass the sieve are placed in another tray separately (trays shown in photo under a)).

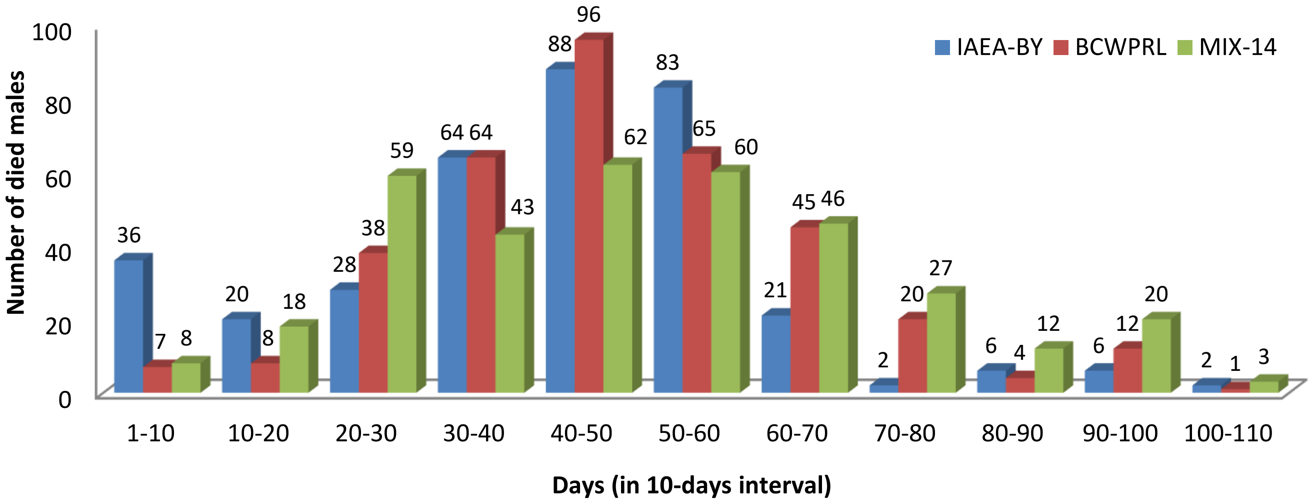

Supplemental Figure S2. Number of died adult males of *Aedes albopictus* recorded in 10 day intervals for three applied larval diet types. Cumulative results obtained from three replicates for each diet are presented.

Supplemental Table S1. Comparison of the water temperature recorded in the rearing trays during the development of *Aedes albopictus* larvae fed by different larval diets.

Tim e	Descriptive Statistics					Univariate test					
	**Diets**	**Mean± Sd**	**Min**	**Max**	**SE**	**SS**	**df**	**MS**	**F**	**SE**	**p**
9h	IAEA BY	26.09 ±1.02	23.50	27.10	0.29	0.02	2	0.01	0.02	0.82	0.98
	BCWPRL	26.03±0.86	24.00	27.30	0.25						
	MIX-14	26.05±0.48	24.90	26.70	0.14						
12h	IAEA BY	25.93±0.95	24.00	27.10	0.24	0.08	2	0.04	0.07	0.77	0.93
	BCWPRL	26.03±0.75	24.90	27.30	0.19						
	MIX-14	25.96±0.58	24.40	26.80	0.15						
15h	IAEA BY	26.45±1.07	23.90	27.80	0.28	0.42	2	0.21	0.31	0.82	0.73
	BCWPRL	26.37±0.84	24.40	27.90	0.22						
	MIX-14	26.21±0.42	25.10	26.90	0.11						

Univariate test was conducted for the significance level α=0.05

Supplemental Table S2. The costs of ingredients (US dollars) used in the recipes of three larval diets for *Aedes albopictus* mass rearing.

Diet mixtures	Ingredients	Ingredients in 100 kg mixtures (kg)	Price of ingredient /kg (USD)*	Cost of ingredient in 100 kg mixture(USD
IAEA-BY	tuna meal	50	0.8	40
	bovine liver	36	63	2268
	brewer's yeast	14	10	100
	vitamin mix	2 g/l solution (4 kg)	28	112
Total				**2520.0**
	bean	16.7	0.6	10.02
BCWPRL	corn	16.7	0.7	11.69
	wheat	16.7	0.2	3.34
	chickpea	16.7	0.8	13.36
	rice	16.7	0.8	13.36
	bovine liver	16.7	63	1052.1
Total				**1103.87**
MIX-14	tuna meal	70	0.8	56
	brewer's yeast	15	10.0	150
	chickpea	15	0.8	12
	vitamin mix	4.6 g/l solution (9.2)	28	257.6
Total				**475.6**

*The presented prices were originally reported by Bimbilé Somda et al. [39, 46] and Khan et al. [45], and were applied for calculation of the costs of 100 kg of each tested diet.
